# Smoking status, dental visits and receipt of tobacco counseling in dental office among mobile and trailer home adolescents

**DOI:** 10.1186/s12903-016-0317-6

**Published:** 2016-11-11

**Authors:** Vinodh Bhoopathi, Huaqing Zhao, Shannon Myers Virtue

**Affiliations:** 1 Department of Pediatric Dentistry and Community Oral Health Sciences, Maurice H. Kornberg School of Dentistry, Temple University, 3223 N Broad Street, Philadelphia, PA 19140 USA; 2 Lewis Katz School of Medicine, Temple University, 3440 N Broad Street, Philadelphia, PA 19140 USA

**Keywords:** Adolescents, Smoking, Mobile and Trailer Homes, Dental Visits, Tobacco Counseling, Access to care, Housing

## Abstract

**Background:**

Mobile and trailer home (MTHs) residents are an understudied group. In this study we determined the cigarette smoking status, dental visits in the past 12 months, and receipt of tobacco counseling in adolescents living in MTHs compared to adolescents living in other types of housing.

**Methods:**

For this secondary data analysis study, we used data of adolescents aged 10 to 19 years (*n* = 74,890) from the 2012 Florida Youth Tobacco Survey (FYTS). Weighted multiple logistic regression model was conducted to understand the differences between adolescents living in MTHs compared to those living in other types of housing.

**Results:**

Approximately 6 % of the sample reported living in MTHs. The regression model showed that older (*p* < 0.0001), female (*p* = 0.0091), and middle school (*p* < 0.0001) adolescents were more likely, and those who identified as Asians (*p* = 0.0006), Black/African Americans (*p* < 0.0001), and Hispanics (*p* < 0.0001) were less likely to be living in MTHs compared to their counterparts. Current established smokers (*p* < 0.0001) and non-established smokers (*p* < 0.0001) were more likely to report living in MTHs compared to non-smokers. Those reporting to have not visited a dental office (*p* < 0.0001) were more likely to be living in MTHs. Those who visited a dental office but not received any tobacco counseling (*p* < 0.0001) were less likely to be living in MTHs compared to their counterparts.

**Conclusions:**

Current cigarette smokers and those not visiting a dental office were more likely to be MTH adolescents. Adolescents reporting to have received tobacco counseling in a dental office were more likely to be living in MTHs.

## Background

In 2013, approximately 17.9 million people [1] lived in 8.5 million mobile or trailer home (MTH) units [2]. While MTH residents comprise a substantial proportion of the US population, very little is known about health behaviors and healthcare utilization in this population. Existing literature on MTH residents focuses primarily on indoor environmental pollution and health issues related to it [3–5]. Other than the data from these small-scale studies, very little is known about MTH residents, particularly adolescents living in MTHs. One of the Healthy People 2020 goals insists on improving the health, safety and well-being of the adolescents [6]. Adolescents are a vulnerable group who may easily adopt unhealthy behaviors as they go through severe hormonal and physical changes [7] and are particularly susceptible to peer influence [8].

Tobacco use is an unhealthy behavior that adolescents may adopt which may lead to long-term harmful health consequences. Data from the Substance Abuse and Mental Health Services Administration for the year 2013 showed that approximately 263,000 12 to 13 year olds and 3.2 million 14 to 17 year olds reported to have used some form of tobacco product in the past year [9]. Smoking early in life may continue into adulthood with evidence showing that smoking during adolescence is a strong predictor of smoking in adulthood [10]. The Agency for Healthcare Research and Quality (AHRQ) published the 2008 clinical treatment of tobacco guidelines, which recommends that the clinicians integrate tobacco cessation and counseling activities into their practice [11]. The United States Preventive Services Task Force (USPSTF) recommends that all clinicians provide appropriate interventions to prevent initiation of tobacco use [12]. The American Dental Association’s (ADA) guidelines related to nicotine use by child or adolescent patient states that a dentist should take the opportunity to ask, provide tobacco cessation counseling, and offer treatment resources [13]. Therefore, it is important that adolescents not only visit dentists but also receive tobacco-related counseling, including preventative counseling, during these visits. There is relatively little data on adolescents living in MTHs in regard to their demographic characteristics, smoking behavior, dental visits, and whether those that have had a visit with a dentist received tobacco counseling.

The aim of this study is to determine whether the following health-related variables are associated with adolescent MTH living status: 1) cigarette smoking status, 2) dental office visits in the past 12 months, and 3) receipt of tobacco counseling from a dentist.

## Methods

### Data source

For this cross-sectional secondary data analysis study, de-identified data from the 2012 Florida Youth Tobacco Survey (FYTS) was used. The FYTS is a state-wide, self-administered, and an anonymous annual survey conducted by the Florida Department of Health targeting middle school and high school students aged 9 to 21 years. For the 2012 FYTS, the sampling included all public middle (6^th^ to 8^th^ grade) and high schools (9^th^ to 12^th^ grade) in all 67 Florida counties. A total of 792 schools were selected; however, only 746 (417 middle and 329 high schools) participated. Class selection was made within the sampling frame using systematic equal probability sampling, with a random start. All students in each selected class were eligible to participate, and a final sample of 75,550 students completed the survey. The analysis for this study was restricted to those aged 10 to 19 years (*n* = 74,890), as defined by World Health Organization as adolescents [14]. The Institution Review Board (IRB) at the Temple University determined that this secondary data analysis study was not a human subject’s research study and that IRB approval was not required.

### Demographic variables

Demographic variables studied were: age (younger adolescents: 10 to 14 years old, and older adolescents: 15 to 19 years old) as defined by the World Health Organization (WHO) [14], gender (male, female), school type (middle school, high school), race (Whites, American Indian/Alaskan Native, Asian, black/African American, Native Hawaiian/Pacific Islander, other), and ethnicity (Hispanic, non-Hispanics).

### Main independent variables

#### Current cigarette smoking status

A new dummy variable (smoking status) on the current cigarette smoking status was derived by using the question: “During the past 30 days, on how many days did you smoke cigarettes?”. This variable categorized the sample into 3 different categories [15]. A smoker was defined as “current established smoker” if someone had smoked one or more cigarettes during 20 or more of the last 30 days, “current non-established smoker” if they smoked one or more cigarettes for less than 20 days of the last 30 days, and a “current non-smoker” for those who did not smoke during the last 30 days. For data analysis purposes, the “current non-smoker” category was used as a reference group.

#### Tobacco counseling in dental office

A question to understand if the adolescent had visited a dental office, and received any advice on the dangers of tobacco during that visit in the past 12 months was included in the 2012 FYTS. The question asked: “Has the dentist or someone in the dentist’s office talked to you about the danger of tobacco use, in the past 12 months?” Three possible responses the adolescents could choose from were: “I have not visited a dentist’s office in the past 12 months”, “yes” and “no”. Those responding that they visited the dental office and also received tobacco counseling were used as the reference group.

#### Outcome variable

Since this study aims to understand the demographic characteristics, smoking status, dental office visits, and receipt of tobacco counseling in the past 12 months in a dental office among adolescents living in MTHs compared to those living in other types of housing, a variable (housing status) was created to categorize the study sample into two groups: those living in MTHs versus those living in other types of housing. This variable was used as the primary outcome measure.

#### Statistical analysis

Univariate and bivariate weighted statistics were conducted. Multiple logistic regression model was conducted, with housing status (MTHs vs other type of housing) as the primary outcome, after adjusting for all main and other independent variables using appropriate weights. Adjusted odds ratios and 95 % confidence intervals were calculated for each association in the regression model, and statistical significance was set at the 0.05 level. We used the SAS 9.3 (SAS Institute, Cary, N.C.) to conduct all statistical analyses.

## Results

The mean age of the sample (*n* = 74,890) was 14.6 ± 2.0 years. Table 1 describes the characteristics of the adolescents aged 10 to 19 years from the 2012 FYTS sample. Slightly more than half of the sample was male (51 %) and older (52 %) adolescents, with 57 % reporting to be in high school. The sample was weighted to represent 54 % Whites, 1 % Native Hawaiian/Pacific Islander, 24 % Black/African American, 2 % Asian, 1 % American Indian/Native American/Alaskan Native, and 18 % other racial categories. Hispanics represented 28 % of the sample. Approximately 6 % (*n* = 4146) reported living in MTHs. The majority of the adolescents were current non-smokers (92 %), followed by current non-established (5.5 %), and current established smokers (2.4 %). Approximately 18 % of the total sample had not visited the dental office in the past 12 months, while 17 % had visited and received tobacco counseling, and 65 % visited but did not receive tobacco counseling (Table 1).

**Table 1:** Descriptive characteristics of the study sample

Variable	*n* ^a^	Percentage
Age		
Younger adolescents	35762	47.9 %
Older adolescents	38951	52.1 %
Gender		
Male	37362	51.0 %
Female	35954	49.0 %
School Type		
Middle	32320	43.4 %
High	42157	56.6 %
Housing		
Trailer or mobile homes	4146	5.6 %
Other types of housing	69684	94.4 %
Race		
White	39699	53.8 %
American Indian/Alaska native	690	0.9 %
Asian	1514	2.0 %
Black/African American	17966	24.4 %
Native Hawaiian/Pacific Islander	511	0.7 %
Other	13398	18.2 %
Ethnicity		
Hispanic	20360	27.8 %
Non-Hispanic	52906	72.2 %
Smoking Status		
Current established smoker	1803	2.4 %
Current non-established smoker	4093	5.5 %
Current Non smoker	68522	92.1 %
Tobacco Counseling in Dental Office		
Did not visit a dental office at all	12705	17.7 %
Visited a dentist office but not receive tobacco counseling	46611	64.9 %
Visited a dentist office and received tobacco counseling	12547	17.4 %

^a^Due to missing values, not all categories add to the total sample size

To understand the differences between adolescents living in MTHs and adolescents living in other types of housing, we conducted bivariate analysis using chi-square tests (Table 2) and then an adjusted multiple logistic regression model using appropriate weights (Table 3).

**Table 2:** Differences in demographic characteristics, cigarette smoking status, dental visits and receipts of tobacco counseling in dental office among adolescents living in MTHs versus not living in MTHs

Variable	Living in Mobile/Trailer Homes	Living in Other types of housing	*p*-value
	*n* (%)	*n* (%)	
Age			
Younger Adolescents	1925 (46.5)	33149 (47.7)	0.15
Older adolescents	2213 (53.5)	36397 (52.3)	
Gender			
Male	2006 (49.8)	34794 (51.0)	0.15
Female	2022 (50.2)	33480 (49.0)	
School Type			
Middle	1871 (45.4)	29778 (42.9)	0.002*
High	2250 (54.6)	39550 (57.1)	
Race			
American Indian/Alaska native	55 (1.3)	629 (0.9)	<0.0001*
Asian	65 (1.6)	1434 (2.1)	
Black/African American	623 (15.2)	17039 (24.8)	
Native Hawaiian/Pacific Islander	36 (0.9)	466 (0.7)	
Other	778 (19.0)	12430 (18.1)	
White	2532 (61.9)	36810 (53.5)	
Ethnicity			
Hispanic	1083 (26.8)	18981 (27.8)	0.19
Non-Hispanic	2951 (73.2)	49313 (72.2)	
Smoking Status			
Current established smoker	318 (7.7)	1464 (2.1)	<0.0001*
Current non-established smoker	427 (10.4)	3627 (5.2)	
Current non-smoker	3377 (81.9)	34234 (92.7)	
Tobacco Counseling in Dental Office			
Did not visit a dental office at all	1082 (27.2)	11486 (17.1)	<0.0001*
Visited a dentist office but not receive tobacco counseling	2130 (53.6)	43929 (65.5)	
Visited a dentist office and received tobacco counseling	763 (19.2)	11620 (17.4)	

*- *p* < 0.05

**Table 3:** Odds of living in MTHs according to adolescent demographic characteristics, cigarette smoking status, dental visits and receipts of tobacco counseling in dental office

VARIABLE	Adjusted OR (95 % CI)	*P* value
Age		
Younger adolescents	0.66 (0.55–0.8)	<0.0001*
Older Adolescents^a^	reference	
Gender		
Male	0.9 (0.83–0.97)	0.0091*
Female^a^	reference	
School Type		
High	0.56 (0.47–0.68)	<0.0001*
Middle^a^	reference	
Race		
American Indian/Alaska native	1.21 (0.90–1.22)	0.2
Asian	0.56 (0.40–0.78)	0.0006*
Black/African American	0.48 (0.42–0.54)	<0.0001*
Native Hawaiian/Pacific Islander	0.98 (0.62–1.55)	0.92
Other	1.08 (0.95–1.22)	0.24
White^a^	reference	
Ethnicity		
Hispanic	0.75 (0.67–0.85)	<0.0001*
Non-Hispanic^a^	reference	
Smoking Status		
Current established smoker	3.81 (3.17–4.60)	<0.0001*
Current non-established smoker	2.08 (1.79–2.42)	<0.0001*
Current non-smoker^a^	reference	
Tobacco Counseling in Dental Office		
Did not visit a dental office at all	1.46 (1.29–1.64)	<0.0001*
Visited a dentist office but not receive tobacco counseling	0.72 (0.65–0.80)	<0.0001*
Visited a dentist office and received tobacco counseling^a^	reference	

*CI* Confidence interval, *OR* Odds Ratio

^a^Reference group, *- *p* < 0.05

A higher proportion of adolescents living in MTHs were current established smokers (7.7 %), and current non-established smokers (10.4 %) compared to those not living in MTHs (Table 2). A greater proportion of those in MTHs (27.2 %) reported not visiting a dental office in the past 12 months compared to those not living in MTHs (17.1 %). Among adolescents who had visited the dentist, a smaller proportion of adolescents living in MTHs reported not receiving tobacco counseling (53.6 %) compared to those not living in MTHs (65.5 %).

The adjusted multiple logistic regression model showed that older (*p* < 0.0001), female (*p* = 0.0091), and middle school adolescents (*p* < 0.0001) were more likely to be living in MTHs compared to their counterparts (Table 3). Self-identified Asians (*p* = 0.0006) and Black/African Americans (*p* < 0.0001) were less likely to be living in MTHs compared to whites. Hispanics (*p* < 0.0001) were less likely to be living in MTHs compared to non-Hispanics. Significant associations were identified in terms of smoking status. Current established smokers were 3.8 times (*p* < 0.0001) the odds of living in MTHs compared to non-smokers. Current non-established smokers were 2.1 times (*p* < 0.0001) the odds of living in MTHs compared to non-smokers. Those who reported to have not visited a dental office were more likely to be living in MTHs compared to those who visited a dental office and received tobacco counseling (*p* < 0.0001). Those who reported visiting the dental office but not receiving tobacco counseling were less likely to live in MTHs compared to those who visited a dental office and received tobacco counseling (*p* < 0.0001).

## Discussion

Approximately 10 % (*n* = 141,102) of the estimated 1.437 million MTH residents in Florida were adolescents (U.S. Census Bureau, Florida Legislature, 2013), indicating that a significant number of adolescents live in MTHs [16]. In this regional study focusing on MTH adolescents living in Florida, we found important and compelling evidence that indicates a need for more studies to understand health behaviors and health care utilization in this group.

Significantly higher proportions (18.1 %) of MTH adolescents reported to be cigarette smokers compared to those living in other types of housing (7.3 %) (Table 2). Additionally, MTH adolescents were significantly more likely to be established and non-established cigarette smokers compared to non-smokers. These findings indicate that cigarette smoking may be an issue among adolescents living in MTHs. Lost dental office visits are missed opportunities for preventing and/or addressing unhealthy habits and identifying preventable diseases, especially among adolescents. Those adolescents who reported not visiting a dental office in the last 12 months were significantly more likely to be MTH adolescents, suggesting that there may be barriers to dental care in this population. However, interestingly, those who reported visiting a dental office but not receiving tobacco counseling were less likely to be MTH adolescents. This means that MTH adolescents that visited a dental office were more likely to receive tobacco counseling. Since adolescents living in MTHs were more likely to report smoking, this may reflect a trend where dentists provide some type of tobacco counseling to adolescents who report smoking or who they perceive to be more likely to smoke. The finding is still encouraging because the dental visit may be an opportunity to provide both dental care and tobacco counseling for this population. However, almost 54 % of adolescents living in MTHs and 65.5 % of adolescents living in other types of housing reported not receiving any tobacco related counseling despite visiting a dental office. These findings suggest that important opportunities to prevent tobacco initiation among adolescents are missed by dentists. Adolescent smoking status, dental office visits, and receipt of tobacco counseling at dental office were significantly associated with housing status. Though literature that directly associates type of housing with health care access and utilization of services is rare, studies have shown that one’s health status may be associated with type of housing. For example, asthma prevalence is significantly higher in children living in public housing compared to private family dwellings [17]. Elderly men in institutional settings are significantly more depressed compared to men in detached homes [18]. Though the theoretical reasoning behind disparities in health care access and utilization based on type of housing is unclear, it may be assumed that people living in better housing units are economically more secure and safe compared to others. However, with very limited information in the literature and from the 2012 FYTS data, it is difficult to make any such assumptions about those living in MTHs and their socioeconomic status.

This study has several limitations. Family income has always been strongly associated with accessing health care services; however, we were unable to include this variable in our analysis because income data is not collected through FYTS. Also, the FYTS did not collect data on medical or dental insurance status and we were not able to determine the influence of insurance on health care visits. Though no other social determinants related to access and utilization of care was available in 2012 FYTS, the specific findings related to MTH adolescents are very unique and compelling. The FYTS collects self-reported data, which might present validity and reliability issues. However self-reported data from adolescents has shown strong validity and reliability even for high-risk behaviors like tobacco and alcohol use. [19, 20]. Despite these limitations, our study has significant strengths. This is the first ever population-based study that demonstrates a negative association between smoking status, dental care utilization and adolescents living in MTHs. And finally, because the participation rate of both middle (77 %) and high school (73 %) students in 2012 FYTS was very high [21], we believe it increases the external validity of the study results.

### Recommendations

Adolescents living in MTHs may be a vulnerable group, and our findings make a convincing case for a more in-depth understanding of their social and physical determinants of health. Improving access to dental care for all adolescents may increase the chance of them receiving tobacco counseling. Possible ways to address these needs may include mobile dental care, community outreach programs in MTH communities, educational campaigns to increase awareness on consequences of tobacco and the importance of utilization of dental care services among MTH residents, and educational campaigns aimed at dental providers increasing awareness of the potential vulnerability of this population. In addition, public health agencies, health care organizatiosn, and policy makers should collaboratively work towards understanding MTH residents, and address their health care needs.

## Conclusions

In conclusion adolescents living in MTHs may be at more risk to engage in cigarette smoking and not visit a dental office. However, MTH adolescents that do visit the dental office may be more likely to receive tobacco counseling. We hope that the results from the study will generate further interest and action in this area and with this population with regard to research, policy, and clinical initiatives.

## Abbreviations

ADA: American Dental Association
AHRQ: Agency for Healthcare Research and Quality
FYTS: Florida Youth Tobacco Survey
MTHs: Mobile and Trailer Homes
USPSTF: United States Preventive Services Task Force
WHO: World Health Organization
